# The death mechanism of the harmful algal bloom species *Alexandrium tamarense* induced by algicidal bacterium *Deinococcus* sp. Y35

**DOI:** 10.3389/fmicb.2015.00992

**Published:** 2015-09-17

**Authors:** Yi Li, Hong Zhu, Xueqian Lei, Huajun Zhang, Guanjing Cai, Zhangran Chen, Lijun Fu, Hong Xu, Tianling Zheng

**Affiliations:** ^1^State Key Laboratory of Marine Environmental Science and Key Laboratory of the Ministry of Education for Coastal and Wetland Ecosystems, School of Life Sciences, Xiamen UniversityXiamen, China; ^2^College of Life Sciences, Henan Normal UniversityXinxiang, China; ^3^Department of Environment and Life Science, Putian UniversityPutian, China

**Keywords:** reactive oxygen species, response of antioxidant enzyme, photosynthetic inhibition, nucleus damage, harmful algal bloom species

## Abstract

Harmful algal blooms (HABs) cause a variety of deleterious effects on aquatic ecosystems, especially the toxic dinoflagellate *Alexandrium tamarense*, which poses a serious threat to marine economic and human health based on releasing paralytic shellfish poison into the environment. The algicidal bacterium *Deinococcus* sp. Y35 which can induce growth inhibition on *A. tamarense* was used to investigate the functional mechanism. The growth status, reactive oxygen species (ROS) content, photosynthetic system and the nuclear system of algal cells were determined under algicidal activity. A culture of strain Y35 not only induced overproduction of ROS in algal cells within only 0.5 h of treatment, also decrease the total protein content as well as the response of the antioxidant enzyme. Meanwhile, lipid peroxidation was induced and cell membrane integrity was lost. Photosynthetic pigments including chlorophyll a and carotenoid decreased along with the photosynthetic efficiency being significantly inhibited. At the same time, photosynthesis-related gene expression showed down-regulation. More than, the destruction of cell nuclear structure and inhibition of proliferating cell nuclear antigen (PCNA) related gene expression were confirmed. The potential functional mechanism of the algicidal bacterium on *A. tamarense* was investigated and provided a novel viewpoint which could be used in HABs control.

## Introduction

Harmful algal blooms (HABs), which almost always destroy the stability of marine ecosystem and damage other marine organisms (Zhang et al., 2014), have broken out more and more frequently in recent years (Anderson et al., 2012). HABs caused by dinoflagellates are the most long-term serious threat to the coastal environment in China (Yang et al., 2012; Li et al., 2014a), and cause great economic loss. *Alexandrium tamarense* belongs to toxic dinoflagellate, which can secrete paralytic shellfish poison, and always form HABs (Zhang et al., 2013). Marine dinoflagellates, as primary producers of the seas and oceans, can be fed by protozoan or other organisms, hence the dinoflagellate toxin can then pass through the food chain and accumulate in organism at the higher trophic levels, and ultimately threaten human health (Li et al., 2015). There are many measures including physical and chemical methods to inhibit HABs (Li et al., 2011; Lee et al., 2013), but these traditional methods are high cost, hard to perform, and especially hazardous to other harmless organisms (Li and Pan, 2013), so biological methods with a reputation for secure and efficient use have been chosen as novel HAB-control methods of regulating blooms (Jeong et al., 2008; Chen et al., 2014).

Biological methods of HAB-control include the utilization of inhibitory effect of marine phytoplankton, protozoans and microorganisms on the growth of HAB algae. Marine microorganisms forming high populations, with powerful function and the most widely distribution (Stocker, 2012), are the first-choice approach to be exploited. Among the microorganisms, bacteria have complex relationships with HAB causing algae, so bacteria with algicidal activity are isolated and identified (Li et al., 2013a,b), and various algicidal bacteria have been used recently to control HABs (Yang et al., 2014). To learn more about the relationships between bacteria and algae, we focused mainly on the reason for the algicidal mechanism.

Previous reports show that the algicidal mechanisms of bacterial inhibition of algal growth involve mainly four aspects, namely cell structure destruction, photosynthesis inhibition, enzymatic activities response, and functional gene expression alteration (Berger and Schagerl, 2004). Marine phytoplankton will produce superfluous reactive oxygen species (ROS) under stress (Mallick and Mohn, 2000), and ROS will destroy the cell structure and photosynthesis, and influence gene expression (Kim et al., 1999). Antioxidant systems including superoxide dismutase (SOD), catalase (CAT), and peroxidase (POD) are induced, but taking a long time to clear ROS (Gill and Tuteja, 2010). The algal cell death mechanism under the function of abiotic factors, such as light (Berges and Falkowski, 1998), temperature (Dunn et al., 2004), or nutrients (Shukla et al., 2008) has been studied for years, but few studies have focused on the molecular mechanisms of the algae in response to biotic stresses, especially the algicidal activity of bacteria.

Our previous research focused on the isolation and identification of algicidal compound secreted by bacterial strain *Deinococcus* sp. Y35, and demonstrates that strain Y35 showed high algicidal activity against *A*. *tamarense* (Li et al., 2015). Herein, algicidal mechanism of strain Y35 on *A. tamarense* were mainly elucidated. The algicidal effect and algicidal procedure were determined using scanning electron microscopy (SEM) and confocal laser scanning microscopy (CLSM). Furthermore, the ROS contents, cell membrane integrity and responses of the antioxidant systems in algal cells were studied. Photosynthetic system inhibition, including pigment contents and photosystem efficiency, were measured. The functional genes expression was also clarified. The ultimate goal of our study was to elaborate the death mechanisms of the algal response to algicidal bacteria, and provide more options for the bio-control of HABs.

## Materials and methods

### Bacterial cultures

*Deinococcus* sp. Y35 (GenBank No. KJ639011) was deposited in Marine Culture Collection of China, with an accession number of MCCC 1F01224. The strain was cultured in Luria-Bertani medium (LB; 10 g of tryptone, 5 g of yeast extract in 1 L of 0.45 μm Millipore-filtered distilled water, pH 7.2–7.6) for 24–48 h and was then preserved at −80°C in LB with 20% (v/v) glycerol.

### Algal cultures

The experimental alga, *A. tamarense*, were deposited in Algal Culture Collection, Institute of Hydrobiology, Jinan University, Guangzhou, China, and the accession number is ATGD98-006. The algal culture was cultivated in f/2 medium prepared with natural sea water, which had been passed through a 0.45 μm filter at 20 ± 1°C under a 12 h: 12 h light-dark cycle with a light intensity of 50 μmol photons m^−2^s^−1^ (Guillard, 1975).

### Analysis of algicidal activity

To determine the algicidal activity of strain Y35 on *A. tamarense*, the fluorescence intensity and color of the algal cultures were monitored. Strain Y35 was inoculated into 20 mL LB medium and grown to the stationary phase at 28°C in a shaker at 120 rpm for 24 h, and then concentrations of 0.5, 1.0, 2.0, and 3.0% of the bacterial culture were added into the algal cultures as treatment groups. At the same time, an algal culture with blank sterile LB or sterile f/2 medium was set up as the control group. The algicidal rate was calculated using the following formula:

$$\begin{array}{l} Algicidal\;rate\left(\text{\%}\right)=\frac{FC-FT}{F\text{C}}\times 100 \end{array}$$

where FT is the fluorescence intensity of treated algal culture and FC the fluorescence intensity of the control algal culture (Zhang et al., 2014).

### Determination of ROS levels

Intracellular ROS was detected using a fluorescent probe, 2′,7′-dichlorofluorescin diacetate (DCFH-DA), based on the reported method (Jakubowski and Bartosz, 2000), with slight modifications. 0.5 mL DCFH-DA (the final concentration in the mixture was 10 mM) was added to the cell particles and the mixture was incubated in an incubator at 37°C in the dark for 1 h with gently blending every 5 min during this time. Then, the cells were collected and washed twice with sterile f/2 medium (without silicate) and finally resuspended in 500 μL f/2 medium. The fluorescence intensity was monitored using a spectrofluorometer with excitation wavelength at 488 nm and emission wavelength at 525 nm.

### Lipid peroxidation and antioxidative enzyme assays of algal cells

After treated with algicidal bacterium for 0, 6, 12, 24, and 48 h, algal cells were pelleted at 1160 × g for 5 min followed by washing in 1 mL of PBS (NaCl 8 g, KCl 0.2 g, Na_2_HPO_4_1.44 g, KH_2_PO_4_ 0.24 g, in 1 L distilled water, 50 mM, pH 7.8) twice, and then homogenized with an ultrasonic cell pulverizer (NingBo Scientiz Biotechnological Co., Ltd, China) 50 times at 80 W (ultrasonic time: 2 s; rest time: 3 s) at below 4°C. Then, the homogenate was centrifuged at 10,000 g for 10 min at 4°C. One milliliters supernatant was used to assay cellular protein which was detected using the “Coomassie brilliant blue protein analysis kit” (Nanjing Jiancheng Bioengineering Institute, China), using bovine serum albumin as the standard. The remaining supernatant were stored at −80°C until they were used to analyze the alteration of malondialdehyde (MDA, a byproduct of lipid peroxidation), and the activities of SOD, CAT, and POD. All the analysis methods followed the kit's operation manual, the samples were mixed with test liquid, and measured by microplate reader after heating with water bath.

### Transmission electron microscopy (TEM)

Algal cells were treated with bacterial culture for 12 h, and were then prepared for TEM. Twenty milliliters of algal culture was collected (1160 × g, 5 min, 20°C), then fixed with 2.5% glutaraldehyde (v/v) for 2–4 h, then washed with 0.1 M PBS (50 mM, pH 7.4) twice. Samples were embedded in araldite resin. Sections (60–80 nm), obtained with an ultramicrotome, were stained in 3% acetic acid uranium-citric acid and viewed using TEM (model JEM-2100HC; JEOL).

### Scanning electron microscopy (SEM)

Bacterial cultures of strain Y35 were added into axenic exponential phase algal cultures at a ratio of 2.0% (v/v). A 10 mL culture was collected (1160 × g, 5 min, 20°C), then fixed in 0.1 M sodium phosphate buffer (PBS, 50 mM, pH 7.4) containing 2.5% glutaraldehyde (v/v) for 2 h and then gently rinsed twice with PBS buffer followed by post fixation in 1% OsO_4_ in the same buffer for 2 h. The samples were then gently rinsed twice with PBS buffer followed by dehydration in a graded ethanol series (30, 50, 70, 90, 95, and 100%) and finally stored in pure tertiary butanol at 4°C overnight. The samples were subsequently critical-point-dried and mounted on stubs. The preparation was sputter coated with gold-palladium at 60:40 and 25:30 nm. The algicidal procedure was visualized and imaged using SEM (model JSM-6390, JEOL).

### Confocal laser scanning microscopy (CLSM)

After treatment with the algicidal bacterial culture, 5 mL of algal cultures were collected at 1160 × g for 5 min followed by washing with 1 mL of PBS (50 mM, pH 7.4). Then DAPI nuclear staining was operated by adding 200 μL DAPI (Beyotime, China) to cover the samples. They were incubated at room temperature in the dark for 5 min, and then the algal cells were resuspended in 1 mL of PBS after washing twice with PBS. A small droplet of algal suspension was placed on a pre-cleaned glass slide and trapped under a coverslip. The algal cells were imaged using CLSM (model Zeiss LSM 780) through a × 401.2 N.A. water immersion objective. DAPI fluorescence was observed through a 435–485 nm band pass filter and chlorophyll fluorescence through a 650–710 nm band pass filter. More than 100 cells from each treatment were examined under a × 40 objective and representative pictures were taken.

### Photosynthetic pigment assay and chlorophyll a fluorescence measurement

Chlorophyll *a* (Chl *a*) and carotenoid were measured spectrophotometrically at 665, 645, and 470 nm after 5 mL of algal culture with or without bacterial culture was extracted with 90% ethanol overnight at 4°C followed by centrifugation at 4°C for 10 min at 8000 g (Inskeep and Bloom, 1985). The pigment contents were calculated using the following equations:

$$\begin{array}{l} {\text{Chlorophyll}\;\text{a}(\text{mg}∕\text{L})=12.7*\text{A}}_{665}\;-\;{2.69*\;\text{A}}_{645} \end{array}$$

$$\text{Carotinoid}(\text{mg/L})=(1000\;*\;{\text{A}}_{470}\;-\;2.05*{\text{C}}_{\text{Chlorophyll a}})/245\;$$

where A_665_, A_645_, and A_470_ represent absorbance values at wavelengths of 665, 645, and 470 nm; and C _Chlorophylla_ represents the content of Chl *a*.

The pulse-amplitude modulation fluorescence measurements were performed using a PAM-CONTROL Fluorometer (Walz, Effeltrich, Germany). Two fluorescence measurements were made: one before (Fo = minimum fluorescence) and one during (Fm = maximal fluorescence) the pulse. The maximum quantum yield (the photosystem II, PS II, photochemical efficiency) was calculated from Fv/Fm (where Fv = Fm-Fo), when the algal cells were dark-adapted for 15 min before measurements under a strong red LED (>3000 μmol m^−2^ s^−1^) (Nymark et al., 2009). The relative electron transport rate (rETR; micromole electrons per square meter per second) was measured (Franklin and Badger, 2001), when eight consecutive light levels of 156, 226, 337, 533, 781, 1077, 1593, and 2130 μmol photons m^−2^ s^−1^ were applied at 10 s intervals. The nonphotochemical quenching (NPQ) levels were measured at the same time. The control, with the addition of 10 μM dichlorophenyldimethylurea (DCMU) was chosen as the positive control.

### RNA extraction and quantitative real-time PCR analysis

Eighty milliliters of algal culture was centrifuged at 1160 × g for 5 min at 4°C after treatment with bacterial culture for 6 and 24 h. RNA was extracted as soon as possible using the RNAiso kit (TaKaRa Company, Dalian, China) following the manufacturer's instructions. For reverse transcription, 1 μg of total RNA was reverse transcripted following the instructions of the PrimeScript RT reagent kit (TaKaRa Company). Real-time PCR was carried out using an SYBR Premix EX TaqTM II kit (TaKaRa Company). The PCR program was: one denaturation step at 95°C for 30 s, 40 cycles of 95°C for 5 s, and 60°C for 30 s, then increasing from 60°C to 95°C by 0.5°C each 5 s. 18S rRNA was used as a housekeeping gene to normalize the expression changes. The relative gene expression among the treatment groups was quantified using the 2^−ΔΔCt^ method.

### Statistics

All data were presented as means ± standard error of the mean and were evaluated using one-way analysis of variance with the three biological replicates followed by the least significant difference test, with *p* < 0.01 and *p* < 0.05 (Origin 8.5 for Windows).

## Results

### Algicidal activity of strain Y35 culture

**Figure 1:**
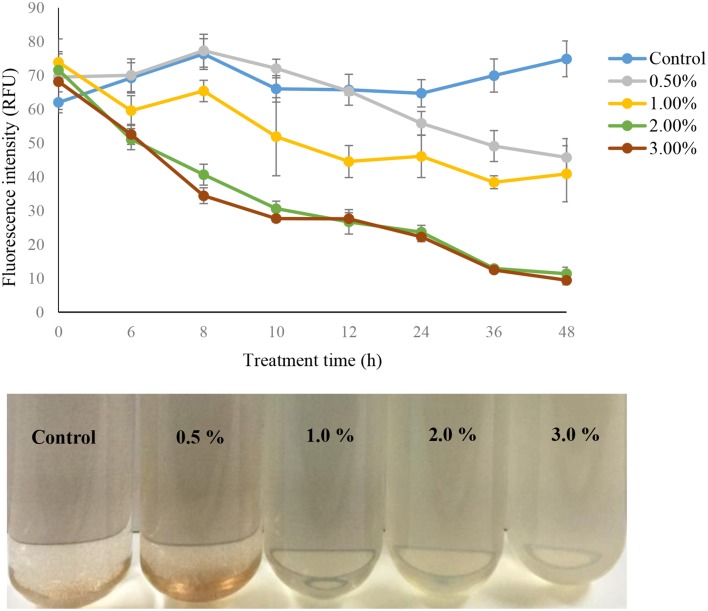
**The algal fluorescence intensity and algal culture of strain Y35 at concentrations of 0.5, 1.0, 2.0, and 3.0% on *A*. *tamarense* at different time points**. All error bars indicate the SE of the three biological replicates.

The algicidal activity of the different concentrations (0.5, 1.0, 2.0, and 3.0%) of strain Y35 cultures was reflected in the fluorescence intensity of the algal cultures (Figure 1). We also compared the fluorescence intensity in the control group with added LB or sterile f/2 medium and the normal algal culture, and there were no obvious differences between them. Therefore, we used the control group with LB medium added as the experimental control group. The fluorescence intensity of algal cultures with concentrations of 1.0, 2.0, and 3.0% showed a significant decrease compared to the control group, but concentration of 0.5% did not exhibit clear differences with the control until 36 h of treatment. Within 48 h of treatment, the algicidal rate of the treatment groups with concentrations of 1.0, 2.0, and 3.0% reached 45, 85, and 87%. Most of the algal cells lysed and died after adding the bacterial culture for 24 h, and the algal culture faded to pale or white color. At the same time, numbers of intact cells were present in the control and the 0.5% concentration treatment group, and the color of the algal culture was brown and normal.

### ROS level and antioxidative enzyme activities under algicidal activity

**Figure 2:**
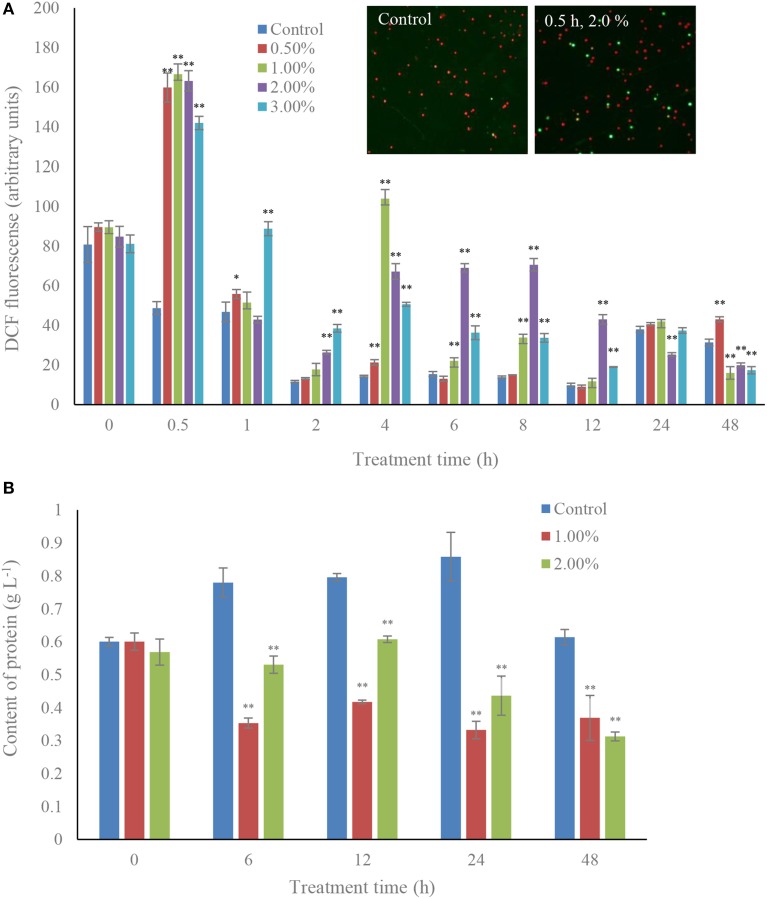
**Effects of the bacterial culture on ROS level (A) and protein contents (B) in *A. tamarense***. All error bars indicate the SE of the three biological replicates. ^*^Represents a statistically significant difference of *p* < 0.05 when compared to the control; ^**^represents a statistically significant difference of *p* < 0.01. Inset represents the green fluorescence of algal cells within ROS between control and treatment groups.

ROS levels were induced under the effect of the algicidal bacteria, and were significantly increased (*p* < 0.01) during the algicidal procedure (Figure 2A). The content of ROS increased after 0.5 h exposure in all the treatment groups, and ROS levels within 0.5 h treatment with concentrations of 0.5, 1.0, 2.0, and 3.0% bacterial culture were 3.28, 3.42, 3.36, and 2.92-fold those of the control. However, the ROS contents in all the treatment groups began to decrease after 1 h exposure until 4 h, when the ROS contents in the concentrations of 1.0, 2.0, and 3.0% rose again, and maintained a high level compared to the control. The protein contents in the 1.0 and 2.0% concentrations decreased greatly (*p* < 0.01) compared to the control (Figure 2B). The trend observed in treatment time was that the protein contents in both these treatment groups presented low levels compared to the control, and the protein contents of the 48 h treatment groups decreased by 40 and 50% of the control at concentrations of 1.0 and 2.0%.

**Figure 3:**
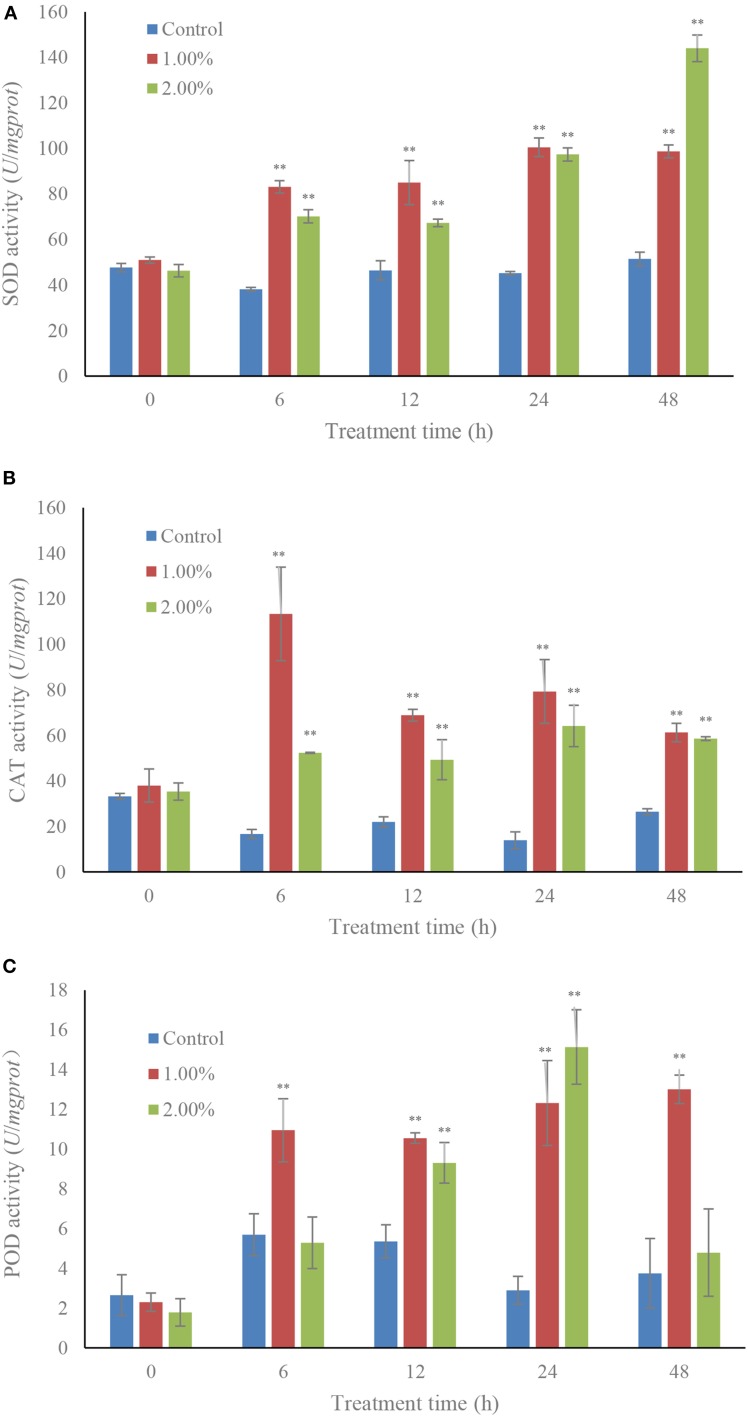
**Effects of the bacterial culture on activities of SOD (A), CAT (B), and POD (C) of *A. tamarense***. All error bars indicate the SE of the three biological replicates. ^**^Represents a statistically significant difference of *p* < 0.01.

The SOD contents increased greatly under the effect of algicidal activity (Figure 3A). The activity of SOD increased gradually with increasing treatment time, and the activity values were 1.92 (*p* < 0.01) and 2.80-fold (*p* < 0.01) those of the control when algal cells were treated with 1.0 and 2.0% bacterial cultures within 48 h. The activity of CAT was obviously increased (*p* < 0.01) in the 1.0% concentration with 6 h of treatment, and then decreased after 12 h of treatment. However, CAT activity in the treatment groups was still much higher than that in the control (Figure 3B). POD activity presented higher levels in the treatment groups than that in the control, and the maximum POD activity was 5.22-fold (*p* < 0.01) that of the control, which was observed within 24 h of exposure to the 2.0% bacterial culture, and then decreased in the 48 h of exposure (Figure 3C). The POD activity in the 1.0% concentration maintained a high and stable level (*p* < 0.01) compared to the control.

### Cellular membrane integrity and the algicidal procedures

**Figure 4:**
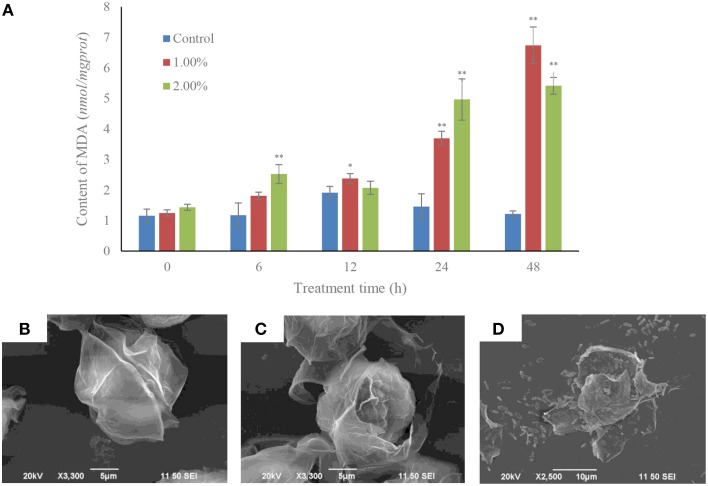
**MDA contents (A) and algicidal procedure (B–D) in algal cells under the effect of the bacterial culture**. **(B)** Control cells of *A. tamarense*; **(C)** a damaged *A. tamarense* cell after 12 h treatment time; **(D)** fragments of death algal cell. All error bars indicate the SE of the three biological replicates. ^*^Represents a statistically significant difference of *p* < 0.05 when compared to the control; ^**^represents a statistically significant difference of *p* < 0.01. Bars **(B,C)** 10 μm, **(D)** 5 μm.

Within 6 h of treatment, the MDA contents were increased (*p* < 0.01) in the 2.0% treatment group compared to the control and 1.0% treatment group. The MDA contents in the 1.0% concentration were induced (*p* < 0.05) after 12 h exposure. After 24 h of treatment, the MDA contents in the 1.0 and 2.0% concentrations were significantly increased (*p* < 0.01) compared to the control. MDA levels after treatment with the 1.0 and 2.0% concentrations for 48 h were 5.55 and 4.46-fold those of the control (Figure 4A).

The algicidal procedures were also determined using SEM, and morphological changes were observed in the *A. tamarense* cells, which were treated with bacterial culture at a concentration of 1.0% (Figures 4B–D). The cell wall and membrane of algal cell in the control retained their integrity, the cell wall with a visible fissure in the middle of the intact cell structure (Figure 4B). However, in the treatment group, cell walls were seriously destroyed, with exposed cell membranes and diffused cytoplasm within the 12 h exposure (Figure 4C). With increased processing time, the algal cells were lysed and part of the cellular substances were decomposed and released from the cells (Figure 4D).

### Effects of the bacterial culture on cellular pigments and photosynthetic efficiency measurements

**Figure 5:**
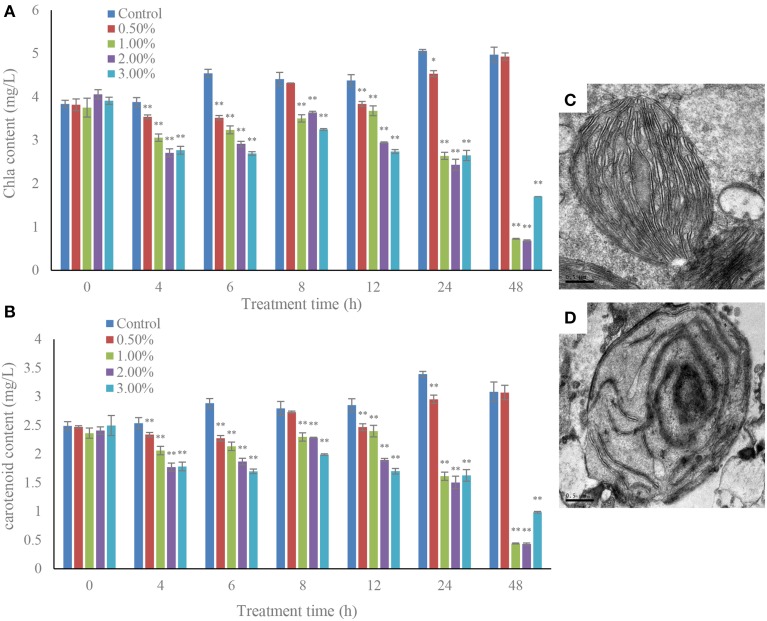
**Inhibitory effects of the bacterial culture on chlorophyll *a* (A) and carotenoid (B) contents in *A. tamarense*, and the TEM morphology of a control (C) and damaged chloroplast (D)**. All error bars indicate SE of the three replicates. ^*^Represents a statistically significant difference of *p* < 0.05 when compared to the control, ^**^represents a statistically significant difference of *p* < 0.01. Bars **(C,D)** 0.5 μm.

The cellular pigments, Chl *a* and carotenoid contents in the algal cells were significantly decreased under the effect at concentrations of 1.0, 2.0, and 3.0% of algicidal culture (Figure 5). The Chl *a* contents decreased (*p* < 0.01) from 4 h of treatment to 48 h (Figure 5A), and the maximum inhibitory effect of the bacterial culture was achieved within 48 h exposure. The Chl *a* contents were approximately 14.6, 13.8, and 34.2% that of the control after exposure to the 1.0, 2.0, and 3.0% concentrations of bacterial culture. However, the 0.5% concentration did not show any obvious effect on Chl *a* content compared to the control. The carotenoid contents in the treatment groups showed similar trends with Chl *a* content, and were greatly decreased during the algicidal procedure (Figure 5B). After 48 h of treatment, the carotenoid contents were significantly (*p* < 0.01) decreased under the effect of the 1.0, 2.0, and 3.0% concentrations of bacterial culture, and were approximately 14.3, 14.0, and 32.0% of the control. Under the algicidal activity of the bacterial culture, the structure and morphology of the chloroplasts changed obviously compared to the control (Figures 5C,D). In the control, the chloroplast structure was intact and clear, with tightly and evenly distributed thylakoids and tubular cristae. However, the chloroplast membrane was destroyed, and the internal structure became sparse and fuzzy, when the chloroplast structure was damaged under the algicidal effect (Figure 5D).

**Figure 6:**
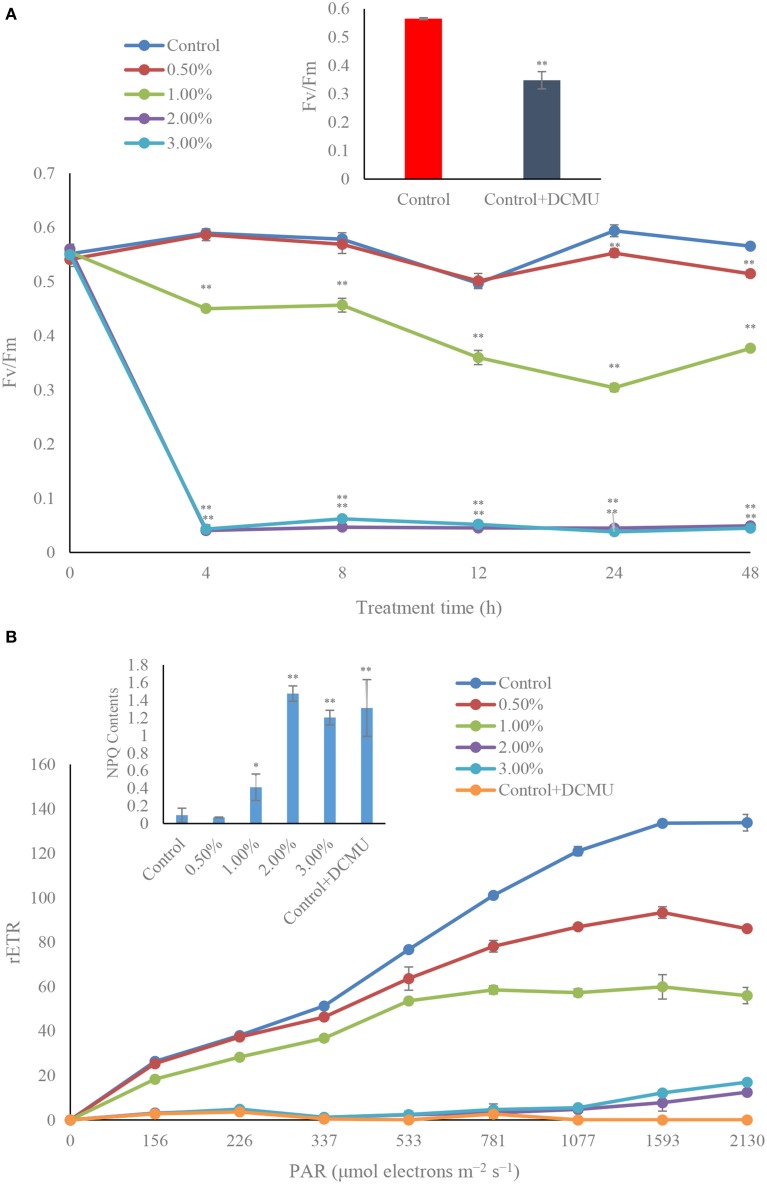
**Photosynthetic efficiency (Fv/Fm) (A), and photosynthetic capacity (rETR) exposed for 4 h (B) of *A. tamarense* treated with different concentrations of the bacterial culture**. All error bars indicate the SE of the three biological replicates. ^*^Represents a statistically significant difference of *p* < 0.05 when compared to the control; ^**^represents a statistically significant difference of *p* < 0.01. DCMU concentration is 10 μM with incubation for 5 min. inset in **(A)** represents the Fv/Fm contents in algal cells after treated by DCMU; inset in **(B)** represents the NPQ contents in algal cells after treated by DCMU.

Investigation of the photosynthetic efficiency in algal cells, Fv/Fm and rETR, revealed that, within 4 h of treatment, the Fv/Fm values were inhibited by the algicidal bacterial cultures, especially the 2.0 and 3.0% concentrations (Figure 6A), where the Fv/Fm value declined to extremely low levels (*p* < 0.01), only 6.8 and 7.3% of the control. In the 2.0 and 3.0% treatment groups, the photosynthetic efficiency was significantly lowered, and the Fv/Fm value was always hold down at a low level. The Fv/Fm value was continuously reduced in the 1.0% treatment group before 24 h, but increased within 48 h of treatment. However, there were no obvious differences of Fv/Fm value between the control and 0.5% treatment groups. We also studied the Fv/Fm value when adding DCMU, and the result showed that the Fv/Fm value was inhibited compared to the control (Figure 6A, inset), which implied that the algicidal bacteria had a similar function with DCMU, and could inhibit the photosynthetic efficiency of the algal cells.

During the algicidal procedure, the value of rETR in the treatment groups decreased obviously (*p* < 0.01) compared to the control (Figure 6B). The rETR values in the 2.0 and 3.0% treatment groups were maintained at a lower level in the 4 h treatment time, compared to the control and the other treatment groups, and had a similar inhibitory effect with DCMU treated. In the 0.5 and 1.0% treatment groups, the rETR values presented a lower level than the control. We compared the NPQ levels in the control and treatment groups (Figure 6B, inset), which implied that the NPQ and DCMU in the treatment groups were much higher than that in the control (*p* < 0.01).

### Effect of the bacterial culture on transcription of the photosynthesis-related genes

**Figure 7:**
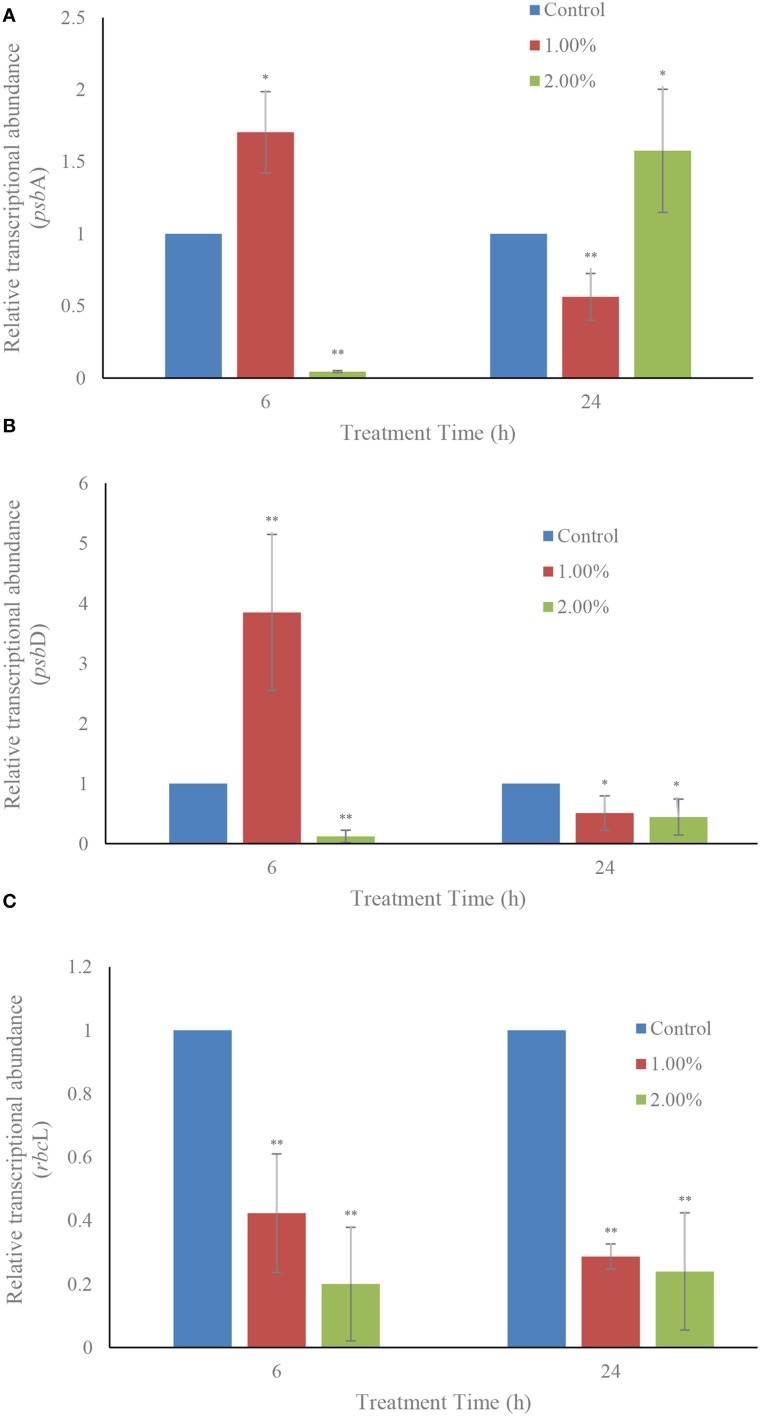
**Effect of the bacterial culture on the transcription of photosynthesis related genes *psb*A (A), *psb*D (B), and *rbc*L (C) in *A. tamarense***. All error bars indicate the SE of the three biological replicates. ^*^Represents a statistically significant difference of *p* < 0.05 when compared to the control; ^**^represents a statistically significant difference of *p* < 0.01.

The effects of the two concentrations (1.0 and 2.0%) of the bacterial culture on transcription of the photosynthesis-related genes, *psb*A and *psb*D (which encode the core proteins of PS II) and the carbon dioxide fixation related gene *rbc*L are shown in Figure 7. Within 6 h of treatment, the relative transcriptional abundances of gene *psb*A were obviously inhibited (*p* < 0.01) in the 2.0% concentration and, at the same time, in the 1.0% concentration had a higher up-regulation value (*p* < 0.05) compared to the control (Figure 7A). However, within the 24 h exposure, the relative transcriptional abundances of this gene showed an opposite result after 6 h of treatment. The gene expression of *psb*A showed down-regulation in the 1.0% and up-regulation in the 2.0% concentration compared to the control. *psb*D exhibited a somewhat similar response to the bacterial culture as *psb*A did (Figure 7B). Within 6 h exposure, the gene expression showed an obvious up-regulation in the 1.0% treatment in contrast with the control, and the relative transcriptional abundances were significantly decreased in the 2.0% concentration. Within 24 h of treatment, the relative expressions of *psb*D were 0.51 (*p* < 0.05) and 0.45 (*p* < 0.05)-fold those of the control at the 1.0 and 2.0% concentrations. Different from the relative transcriptional abundances of gene *psb*A and *psb*D, the transcription of *rbc*L was inhibited in all treatment groups in both the 6 h and 24 h exposure (Figure 7C). The 1.0 and 2.0% treatment groups showed obvious inhibitory effects (*p* < 0.01) on the transcription of *rbc*L after 6 h of treatment. The transcription of *rbc*L in the 1.0 and 2.0% treatment groups was only 0.29− (*p* < 0.01) and 0.24-fold (*p* < 0.01) compared to the control within the 24 h exposure.

### Effects of the bacterial culture on the nuclear system

**Figure 8:**
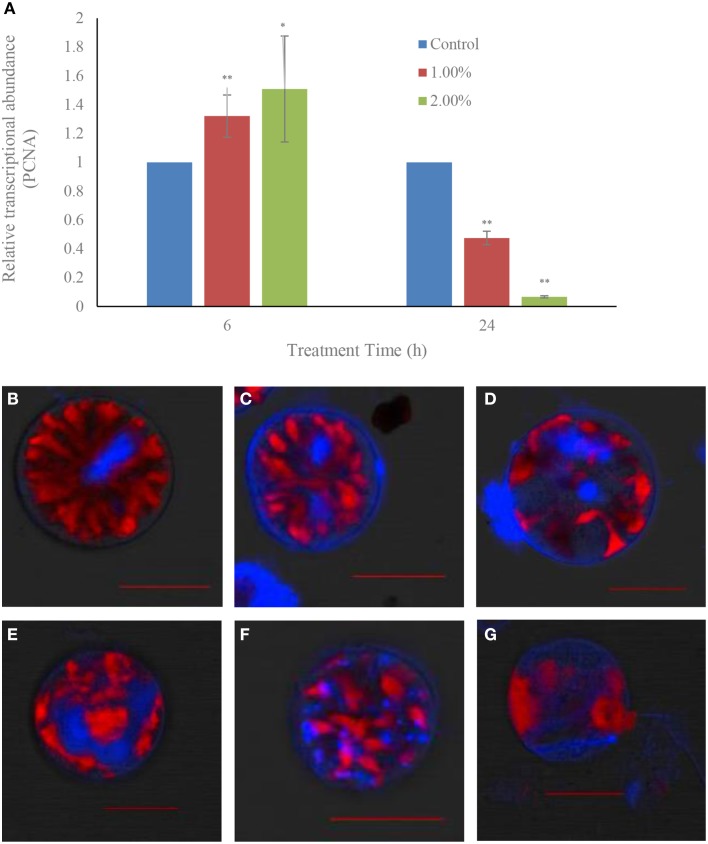
**Effect of the bacterial culture on the transcription of the PCNA gene (A) and the destruction of nuclear structure (B–G) in *A. tamarense***. **(B)**, control cells; **(C–E)**, 12 h treatment time; **(F,G)**, 24 h treatment time. Red fluorescence is algal chloroplast fluorescence, which represents the strength of algal vitality; blue fluorescence represents the nuclear area. Scale bar = 20 μm. All error bars indicate the SE of the three biological replicates. ^*^Represents a statistically significant difference of *p* < 0.05 when compared to the control; ^**^represents a statistically significant difference of *p* < 0.01.

Within 6 h of treatment, there were no inhibitory effects of the bacterial culture on the gene expressions of the proliferating cell nuclear antigen related gene (PCNA): the gene expression showed up-regulation in both the 1.0 (*p* < 0.01) and 2.0% (*p* < 0.05) treatment groups compared to the control (Figure 8A). However, the inhibitory effect on gene expressions showed up in the 24 h exposure in both the 1.0 and 2.0% concentrations. The relative transcriptional abundances of gene PCNA were significantly decreased (*p* < 0.01) in the 1.0 and 2.0% treatment groups within the 24 h treatment time, and were only 0.47 (*p* < 0.01) and 0.07-fold (*p* < 0.01) those of the control.

Degradation of the nuclear structure was also observed using CLSM (Figures 8B–G). In the control, the normal growth cells had a rod-like, clear, compact and stable nuclear structure, and all the genetic material which was stained with DAPI were confined to the nuclear area by the nuclear membrane (Figure 8B). After treatment for 12 h with the 1.0% concentration, the nuclear structure was changed and became varied and dispersed, and the DAPI fluorescence could be seen in the different areas of the algal cells, and was obscure and weak (Figures 8C–E). With prolonged treatment time, the nuclear material was dispersed within the whole cells, and showed a broken and diffused state (Figure 8F). Finally, the DAPI fluorescence could almost not be observed, the cells were lysed and lost their chloroplast fluorescence (Figure 8G).

## Discussion

Algicidal bacteria have been studied for years as one of the most safety and effective ways to implement HAB bio-control (Imai et al., 1993; Skerratt et al., 2002; Mayali and Azam, 2004), but the functional mechanism is still not clear. To explore the algicidal mechanism and death procedure of HAB causing algae under algicidal bacteria treatment, further work were carried out here, including the algicidal activity and procedure observation, ROS contents and antioxidative enzyme activities assays, photosynthetic efficiency and photosynthesis-related gene expression determination, and the cell nuclear system integrity analysis.

In the last few decades, many studies have reported the existence of algicidal bacteria with antagonistic activity on marine phytoplankton. The algicidal bacteria may increase in abundance concurrently with the decline of algal blooms, suggesting they may affect algal bloom dynamics (Yang et al., 2012). Therefore, bacteria with algicidal activity could be used to control HABs, and many algicidal bacteria have been isolated and applied. In our study, one algicidal bacterium *Deinococcus* sp. Y35 was used to regulate the growth of the toxic HAB causing alga *A*. *tamarense*. The bacterial culture of algicidal bacteria was added into algal cultures at different concentrations and the fluorescence intensity of the alga was used to reflect the algal biomass. The fluorescence intensity of algal cells in high concentrations of the bacterial culture dropped obviously compared to the control and 0.5% treatment groups, which implied that strain Y35 had a notable algicidal effect on *A*. *tamarense*, since the effect began when the concentration reached more than 1.0% and the higher concentrations were even more efficient. Fluorescence intensity was used as an index to reflect algal growth status, and the linearity between the algal cell number and fluorescence intensity was certified by Zhang et al. (2014). Many algicidal bacteria have been isolated, *Streptomyces* sp. RPS has an algicidal effect on the HAB species *Phaeocystis globosa* (Zhang et al., 2014), and *Cytophaga* sp. 41-DBG2 is an algicidal bacterium which is antagonistic to the HAB dinoflagellate *Karenia brevis* (Mayali and Doucette, 2002). Although many algicidal bacteria have been reported at present, more knowledge on the algicidal mechanism was still needed in order to conduct extensive research.

ROS are highly reactive and toxic to live cells, including ${\text{O}}_{2}^{-}$, H_2_O_2_, ^1^O_2_, HO_2_, OH, ROOH, ROO, and RO, which cause damage to proteins, lipids, carbohydrates and DNA, and ultimately result in cell death (Gill and Tuteja, 2010). ROS in algal cells are produced continuously as byproducts of various metabolic pathways that are localized in different cellular compartments, such as the chloroplast, mitochondria, and peroxisomes. Superfluous ROS is produced when the algal cells are under stress (Asada, 2006). To scavenge ROS and relieve the degree of damage, antioxidant enzyme systems such as SOD, CAT, and POD in the algal cells are activated and employed. SOD can lead to the dismutation of ${\text{O}}_{2}^{-}$ to H_2_O_2_ and then its decomposition convert them into H_2_O and O_2_ under the help of CAT and POD, to ease the oxidative stress. Heavy metals are toxic to marine algae including breaking the dynamic balance of cellular redox status through the production of ROS. Algal antioxidant systems can respond to heavy metals by enhancing the activities of the antioxidant systems. However, high levels of metal pollutants are fatal to algal cells because higher ROS levels exceed the capacity of the cell to cope (Pinto et al., 2003). Algicidal actinomycetes can induce ROS production in algal cells and the response of the antioxidant system finally causes oxidative damage to the algae cells (Zhang et al., 2013). We studied DCF fluorescence and the antioxidant enzyme activities, results showed the ROS contents were significantly increased in the treatment groups compared to the control within 0.5 h of exposure, which implied that excess ROS were produced in the algal cells under the stress from the algicidal bacterium. We also observed that the ROS contents decreased obviously to the control level except in the 3.0% treatment group within a 1 h treatment time, and the ROS contents in the treatment groups fluctuated during the exposure time, whereas the ROS contents in the control always remained at a low level. Although ROS contents declined in the treatment groups, and could be eliminated by the antioxidant systems, they were much higher than that in the control. These illustrated that high concentrations of the bacterial culture induced ROS overproduction in the algal cells, which could be relieved by the algal antioxidant systems. However, once oxidative stress exceeded the resistance effects of the algal cells, oxidative damage occurred.

To respond to the oxidative stress from ROS, the antioxidant enzyme systems including SOD, CAT, and POD in algal cells were activated (Figure 3). The quick response of SOD and CAT activities meant that the ROS contents had been converted into H_2_O_2_ and then decomposed completely. The POD activities did not show obvious increase over a short time, but were obviously increased (*p* < 0.01) compared to the control after being treated for 12 h with concentrations of 1.0 and 2.0%. However, the POD activities in the 2.0% treatment group returned to the normal level after a longer time exposure of 48 h, which implied that POD activities were used to relieve the superfluous ROS caused by high concentrations of bacterial culture. Antioxidant enzyme systems are widely used for protection against ROS damage in algal cells. Metal-induced lipid peroxidation and the response of antioxidant enzymes is reported in the marine microalga *Pavlova viridis*, where enhanced lipid peroxidation is notable with the increase of metal concentrations and total SOD and CAT activities were enhanced in response to copper and zinc, respectively (Li et al., 2006). The response of antioxidant enzymes in the marine diatom *Phaeodactylum tricornutum* to cope with copper toxicity showed that SOD and CAT activities increased a few hours after Cu addition, suggesting that CAT is the major enzyme for scavenging H_2_O_2_ (Morelli and Scarano, 2004). Although the antioxidant enzyme systems in algal cells are always enhanced by environmental stress, the ROS induced by algicidal bacteria are also a main factor resulting in responses of antioxidant enzyme systems. In our study, the bacterial culture of algicidal bacteria induced ROS overproduction, and then activation of some antioxidant enzymes was enhanced to counteract the oxidative stress; SOD and CAT activities were confirmed to be the main enzymes for relieving ROS damage.

The cell membrane is the cell barrier to prevent extracellular substances entering into cells and guarantees that the intracellular environment remains relatively stable, and the biochemical reaction is promoted. MDA is used to reflect the degree of lipid peroxidation and is also an indicator of cellular oxidative damage (Siripornadulsil et al., 2002). The toxic impacts of lead, copper and zinc increase the MDA contents in the cyanobacterium *Spirulina platensis*-S5 (Choudhary et al., 2007), and it is also certified that the MDA level is significantly increased after a short exposure to allelochemical (Qian et al., 2009). The high level of MDA indicated that the algal cell membrane was damaged by the algicidal activity of strain Y35, and so cell membrane integrity was lost. The SEM images showed the algal cells gradually lost their intact cell structure. The cell wall and membrane were broken, and intracellular substances spilled out of the cell. Therefore, we deduced that the cell membrane lost its integrity, and the destruction of cell structure was confirmed.

Photosynthetic pigments in dinoflagellates include mainly Chl *a* and carotenoid, which participate in absorption, transmission of light energy, or cause primary photochemical reactions in the process of photosynthesis. Photosynthesis is catalyzed by two major pigment-protein complexes, Photosystem I (PS I) and Photosystem II (PS II) (Nymark et al., 2009). It is well known that the photosynthetic apparatus, particularly the PSII complex, is sensitive to adverse environmental pressure such as strong light (Styring et al., 1990), and low and high temperature (Terzaghi et al., 1989). Therefore, the photosynthetic pigment contents were measured. Fv/Fm which represents the PSII efficiency, rETR which represents the maximum amount of electrons generated in PSII (Consalvey et al., 2005) and NPQ which reflects photoprotection ability were determined, and the *psb*A and *psb*D genes, encoding the D1 and D2 proteins that together form the core of the PSII reaction center (Marder et al., 1987), and the *rbc*L gene which codes for the large subunit of ribulose-1,5-bisphosphate carboxylase/oxygenase (Chase et al., 1993) were studied. The results showed that the structure of the chloroplast was seriously damaged, and the chloroplast membrane was broken down, as well as the internal structural integrity. Photosynthesis depends on the absorption of sunlight by the photosynthetic pigments, and so the decline of the pigment content inevitably influences the normal operation of the photosynthetic system. The inhibitory effect on Fv/Fm values indicated that the algal photosynthetic efficiency was seriously damaged, and had lost its normal function. DCMU treatment was also shown to strongly reduce Fv/Fm values, which implied that the bacterial culture showed a similar function with DCMU in inhibiting photosynthesis (Figure 6B). The rETR values were greatly inhibited in the treatment groups, especially with high concentrations of bacterial culture, and DCMU additions completely inhibited the electron transfer of the algal cells. In the presence of DCMU, the electron transport chain was blocked, and the bacterial culture showed the same damage effect with DCMU, which implied that algicidal bacteria could destroy electron transfer and influence the maximum amount of electrons generated. The NPQ values were significantly increased (*p* < 0.01) in high concentrations of bacterial culture and with DCMU additions compared to the control, which illustrated well the photosynthetic stress which induced by algicidal activity. A secondary metabolite from *Nostoc XPORK*14A not only reduces net photosynthetic activity and significantly modifies the electron transport properties of PS II in *Synechocystis* PCC 6803, but also inhibits photosynthesis and growth of the algal cells (Shunmugam et al., 2014), moreover, the accumulation of viral glycosphingolipids in algal cells is accompanied by a reduction in cell abundance, and a severely compromised photochemical quantum yield of PSII (Vardi et al., 2009). Thus, algal cell death is closely linked with photosynthesis, and bacterial cultures inhibit algal photosynthetic efficiency, eventually cause algal cell death.

The relative transcriptional abundances of *psb*A and *psb*D showed a similar result to each other: gene expression in the 1.0% treatment group were enhanced in the first treatment time, but was significantly inhibited (*p* < 0.01) as time goes on. We speculated that the reason for the enhancement was the self-repair ability of PS II in algal cells (Ham et al., 2010). However, gene expression was eventually suppressed with increased processing time. The *psb*A gene expression was obviously inhibited (*p* < 0.01) in the 2.0% concentration compared to the control within 6 h of treatment, and showed up-regulation (*p* < 0.05) within 24 h of treatment. Higher concentrations of bacterial culture had a stronger algicidal activity, and the algal self-repair effect could not counteract the algicidal stress in a short period of time, so that gene expression was inhibited in the beginning. Alongside exposure, the algal self-repair ability relieved the algicidal effect, and avoided inhibition of the gene expression. The transcript abundances of *psb*D in the 2.0% treatment group was always down-regulated during the exposure, implying that bacterial culture inhibited *psb*D gene expression, and had a relationship with the inhibition of photosynthesis. The transcript abundances of *rbc*L in the 1.0 and 2.0% concentrations were significantly down-regulated (*p* < 0.01) within the 6 h and 24 h of treatments (Figure 7C). When the transcript accumulation for the *psb*A, *psb*D, *psb*D-C, *rbc*L-S, and *rrn* genes in *Synechocystis* 6803 is followed under different light conditions, the relative abundance of these transcripts differ between high and low light conditions (Mohamed and Jansson, 1989). Our results illustrated that algicidal activity seriously inhibited photosynthesis-related gene expression.

The nucleus, which is the most important structure of algal cells, is the genetic information database and the control center of cell metabolism, playing an important role in cell metabolism, growth, and differentiation. To understand whether cell nucleus integrity was influenced by the algicidal activity, the PCNA gene expression and structure of nucleus were determined (Figure 8). The gene expression of PCNA did not show obvious distinction between control and treatment groups within 6 h of treatment, but the gene expression was greatly inhibited (*p* < 0.01) in treatment groups compared to the control within 24 h exposure. The PCNA gene has a close relationship with DNA synthesis, plays an important role in cell proliferation and reflects the state of cell proliferation (Liu et al., 2005). PCNA gene expressions were inhibited by algicidal activity, which implied that the algal cell nucleus was genetically influenced. As shown in Figures 8B–G, the morphology and structure of the cell nucleus changed alongside the algicidal procedure. In summary, the cell nucleus system was seriously damaged under the action of algicidal bacteria.

In conclusion, the algicidal bacterium *Deinococcus* sp. Y35 efficiently inhibited the growth and photosynthesis of the HAB species *A*. *tamarense*. Under this algicidal activity, superfluous ROS was induced, the antioxidant enzyme systems were activated, cell membrane integrity was lost, furthermore, the photosynthetic system including photosynthetic pigments, photosynthetic efficiency and photosynthesis-related gene expression were seriously damaged, and cell nucleus systems were destroyed. Destruction of cell structure, inhibition of critical functions and damage of the core systems ultimately caused algal cell death.

### Conflict of interest statement

The authors declare that the research was conducted in the absence of any commercial or financial relationships that could be construed as a potential conflict of interest.
